# Supplementary material for: Learning curve and surgical outcome of robotic assisted colorectal surgery with ERAS program
Source: Sci Rep. 2022 Nov 29;12:20566. doi: 10.1038/s41598-022-24665-w (PMC9709162; doi:10.1038/s41598-022-24665-w)
Supplement: Supplementary file 2 — Supplementary Information 2. [file 41598_2022_24665_MOESM2_ESM.zip › RAL Raw data-Ñ[▒K/138/OP N_ñw╝╨░O▒Kñσ.pdf]

W83 / W83

臺中榮民總醫院
手術室護理記錄

[redacted]

基本資料

- 室別： 開刀房-17室，手術科別： CRS，手術類別： 常規，日期： 2021/12/30
- 進入時間： 1540 ； 麻醉開始時間： 1600 ； Time Out時間： 1750 ； 劃刀時間： 1752 ； 手術結束時間： 2325 送出時間： 2340 ；
- 麻醉方式： GE

  診斷： Malignant tumor of rectum(12/14心導管,12/10手術先延期)

  手術名稱： Robot Assisted Low Anterior Resection

  手術局部用藥： 無

  術前預防性抗生素：
  - 抗生素名稱一： Veterin 1GM IVD由麻醉護理師執行
  - 時間： 1705
  - 上次簽章: 許臺真

  ，追加預防性抗生素：
  - 抗生素名稱一： Veterin 1GM IVD由麻醉護理師執行
  - 時間： 2125
  - 上次簽章: 邱欐婇
- 手術醫師： 蔣鋒帆，陳瑨夫，陳怡豪
- 麻醉醫師： VS 謝昀叡
- 刷手護士A ： 林貝臻，時間： 1540，刷手護士B ： 張佑禎，時間： 1540，接替刷手一： 李郁萱，時間： 1800，接替刷手二： 林家羽，時間： 2300

  流動護士A ： 許臺真，時間： 1540，接替流動一： 賴芸，時間： 1700，接替流動二： 許臺真，時間： 1730
- 更多接替及流動護士： 接替流動三： 邱欐婇，時間： 2000，接替流動四： 詹佳穎，時間： 2200，接替流動五： 陳心慈，時間： 2300

皮膚狀況

A-V Shunt： 無

術前： 完整

記錄者： 上次簽章: 許臺真

術後： 完整

記錄者： 上次簽章: 陳心慈

手術準備

手術臥式 單一手術臥式

-
  - 手術臥式： 截石臥式
  - 使用附件： 溫毯 8 ℃
  - 皮膚準備： Hibiscrub，2% Chlorhexidine
  - 電燒： 使用記錄者： 上次簽章: 許臺真

止血帶T： 無

植入物

- Davinci Endo GIA45mm 綠釘*2

  GIA 80-3.8mm*1/colon

，記錄者: 上次簽章: 陳心慈

標本送檢

- 細菌培養 無
- 細胞學 無
- 冰凍切片 無
- 病理標本

  組織名稱一： dista cut end

  ，

組織名稱二： IMA Root

,

組織名稱三： rectum

- 截肢 無

標本交付 標本： 1 袋 ，標本： 0 盒 ，標本： 2 瓶 ，細胞學： 0 管 ，Culture Tube： 0 管 ，被交付醫師： CR 陳瑢夫，

- 記錄者： 上次簽章: 邱欐嫊
- 時間： 2200

傷口術後敷料

傷口敷料： 傷口敷料： 無

- 請於圖上標示部位：

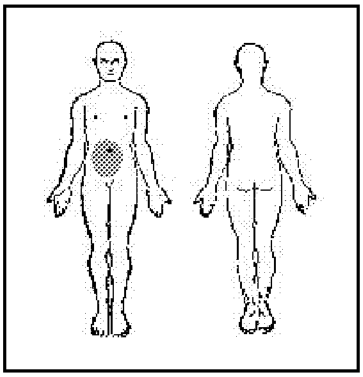

- 傷口敷料： 紗布

導管

導管	天數	導管資訊	插入日期	插入時間	更換日期	拔除日期	拔除時間	拔除原因	預計拔除/更換	預計拔除/更換日期
Foley(1)	2	號碼:16 Fr.; 部位:膀胱;	2021/12/30	1615						
A-line (1)	2	號碼:20 G; 部位:左手;	2021/12/30	1620						
Double Lumen CVC(1)	2	號碼:8 Fr; 深度:15cm; 部位:jugular (右); 象限:RU;	2021/12/30	1630						

用物計數

- 手術中出血量： 少 c.c.
- 手術中尿量： 400 c.c.
-
    - 其它： 無

用物計數： 單一切口

- 1.器械： 正確，2.縫針： 正確，3.刀片： 正確，4.紗布： 正確，5.腹部墊： 未使用，6.紗球： 未使用，7.PEANUTS ： 未使用，8.紗條： 未使用，9.絲帶： 未使用，10.針頭： 未使用，11.Vessel loop： 未使用，12.Boots： 未使用，13.Jelco： 未使用，14.棉條： 未使用，
    - 第一次：
        - 刷手護士 ： 李郁萱
        - 流動護士： 上次簽章: 邱欐嫊
        - 時間： 2120

    ,
    - 第二次：
        - 刷手護士 ： 李郁萱
        - 流動護士： 上次簽章: 邱欐嫊
        - 時間： 2130

    ,
    - 第三次：
        - 刷手護士 ： 李郁萱
        - 流動護士： 上次簽章: 邱欐嫊
        - 時間： 2200

備註

備註： NIL

- 記錄者： 上次簽章: 許臺真
- 時間： 1730

填寫護理人員 陳心慈

正式摘要

W83 / W83

doctorRank:B
